# Lyophilized bovine acellular tendon linear fiber material for the reconstruction of attachment structure of paraspinous muscles: an animal in vivo study

**DOI:** 10.1007/s10856-022-06701-3

**Published:** 2022-12-03

**Authors:** Bo Yuan, Yi-fan Tang, Zheng Xu, Jun-cheng Wang, Sheng-yuan Zhou, Xiong-sheng Chen

**Affiliations:** Spine Center, Department of Orthopedics, Shanghai Changzheng Hospital, Naval Medical University (Second Military Medical University), Shanghai, 200003 China

## Abstract

Low back pain is common after lumbar spine surgery and the injury from extensive detachment of paraspinal muscles during the surgery may play a vital role. Previously, we prepared a bovine acellular tendon fiber (ATF) material through lyophilization and proved that it could retain its original fibrillar structure and mechanical properties. The objective of this study is to evaluate the effectiveness of this new fiber material used for attachment structure reconstruction of paraspinal muscle. Defect of spinous process, interspinous and supraspinous ligament was established on lumbar spine in rabbit and rat and ATF linear material was implanted to reconstruct the attachment structure. Ultrasound showed the cross-sectional area of the paraspinal muscle in ATF group was larger than that of control group in rats. MRI showed the irregular shape and high signal changes in control group, but regular shape and uniform signal in the ATF group in rabbit. For Electromyogram, the frequency of evoked potential in control group was lower than ATF group and normal rats. HE and Masson staining showed good tissue healing, and immunohistochemical results showed the immune rejection of ATF is significantly lower than that of suture. Reconstruction of the attachment structure of paraspinous muscles with ATF linear material could maintain the morphology, volume and function of paraspinal muscle. ATF material has the potential to be used to manufacture personalized ligaments and other tissue engineering scaffolds.

**Figure:**
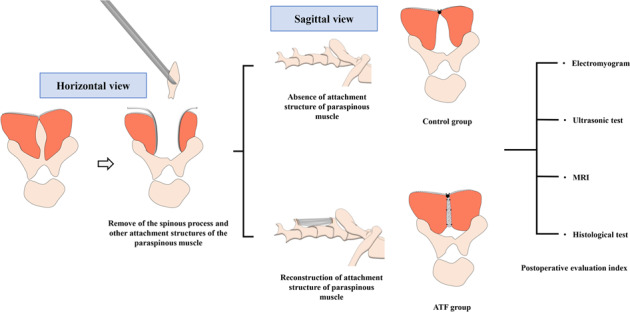
Graphical abstract

Graphical abstract

## Introduction

Posterior lumbar surgery is one of the major approaches to deal with lumbar spine disorders. Low back pain after lumbar spine surgery is considered to be a common clinical disease, which seriously affects the recovery of patients [1–3]. The main possible source of pain is the lesions of the paraspinal muscles, with muscle detachment and retraction during the surgery that may potentially result in crush injury from retractor, devascularization, and denervation [4–6].

The posterior exposure process of the lumbar spine is closely related to the injury of the paraspinous muscles [7, 8]. The reasons are as follows: (1) Paravertebral muscles become fvcompressed by self-retaining retractors at high pressures during posterior lumbar surgery, resulting in ischemic damage and electrophysiological abnormalities, which has been proved in animal and human patients [9]; (2) The attachment structure of paraspinal muscles, the lamina, spinous process, interspinous process and supraspinous ligament were removed during the surgery. Continuous contraction leads to increased intramuscular pressure and chronic ischemic atrophy of the paraspinous muscles after surgery [10]; (3) Direct damage to the paraspinous muscles, such as muscle-splitting and retraction may lead to local inflammation and decreased trunk muscle strength caused by multifidus muscle atrophy [11]. Among them, extensive detachment of paraspinal muscles plays a vital role in the development of low back pain, reduced trunk muscle strength and late-onset spinal instability after posterior lumbar surgery.

Theoretically, intraoperative reconstruction of the attachment structure of the paraspinal muscles is an important measure to prevent the continuous contraction and atrophy of the paraspinal muscles after the operation. It is of great significance for the prevention of low back pain after lumbar surgery, postoperative rehabilitation and recovery. Wu et al. studied the effectiveness of the repositioning suture of the erector spinae muscle for lumbar spine surgery, and they found the novel method could markedly lessen low back pain compared with conventional suture method [12], but the procedure performed required an intact spinous process, which was unsuitable for laminectomy or posterior spinal fusion procedures. However, current literature about directly reconstructing the paraspinous muscles is rare. To the best of our knowledge, effective autologous or allogeneic tissue to reconstruct the attachment structure of the paraspinal muscles has not been reported until now.

In previous study [13], we prepared a lyophilized bovine acellular tendon fiber (ATF) material and proved that it could retain its original fibrillar structure and mechanical properties. We hypothesized that lyophilized ATF linear material may provide the possibility of preparing personalized ligament and other tissue engineering scaffolds. In this study, we prepared the lyophilized ATF, then twisted into linear materials by hand. Defect of spinous process, interspinous process and supraspinous ligament was established on lumbar spine in rabbit or rat and ATF linear material was implanted to reconstruct the attachment structure. Though the in vivo study, we found the reconstruction of the attachment structure of paraspinous muscles with ATF linear material could maintain the morphology, volume and function of paraspinal muscle. This work suggested ATF material has the potential to be used to manufacture personalized ligaments and other tissue engineering scaffolds.

## Materials and methods

### ATF linear material preparation

The ATF preparation method was modified and finished through lyophilization and decellularization, which was reported in our previous study [13]. Briefly, the middle section of extensor and flexor tendons of the limb from adult bovines were harvested within 8 h of slaughter. The loose connective tissue and fat attached to the outside of the tendon were removed, then the tendon was dissected longitudinally and stored at −20 °C. The frozen tendons were lyophilized for 24 h at −56 °C under vacuum (VirTis BENCHTOP, USA). The lyophilized tendons were separated longitudinally to expose the inner tendon fibers, and tendon fiber bundles were manually separated further. Then, the bundles were decellularized as follows: (1) immersed in tris buffer (10 mM) with serpin for 12 h. (2) treated with 0.05% trypsin at 37 °C. (3) submerged in Hanks’ buffer containing 90 U/ml deoxyribonuclease (type II from bovine pancreas) for 2 h at 37 °C. (4) processed with ultrasonic processor (Qsonica Q700, USA) for 5 min and stored with mycillin solution at −4 °C.

The ATF bundles were centrifuged (CNTS-C01, China, 8000 rpm) into strips as the study we reported before [13], then humidified with double-distilled water and unidirectionally twisted into a linear material by hand (Supporting Information 1).

### Animal model establishment and material implantation

#### Rabbit

**Fig. 1:**
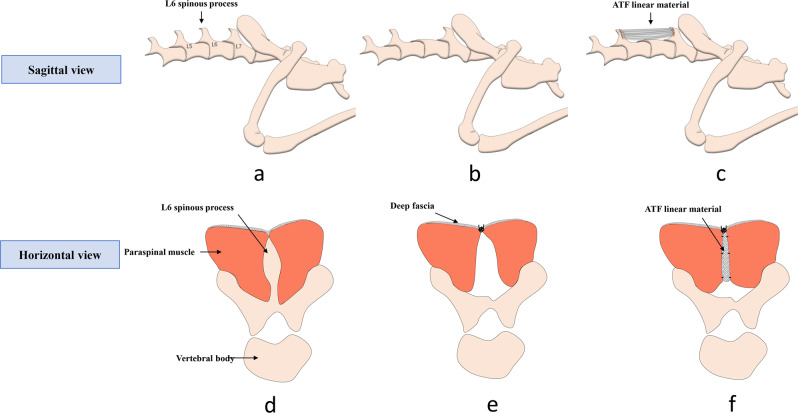
Rabbit model establishment and material implantation. **a** Lumbar sacral bone structure; **b** Resection of L6 spinous process; **c** Use AFT linear material to fix between L5 and L7; **d** cross-section of paraspinal muscle near lumbosacral spinous process; **e** Direct suture of bilateral deep fascia after resection of the interspinous ligament; **f** Bilateral paraspinal muscles was sutured to fix on the AFT linear material between L5 and L7 spinous process

Ten male New Zealand rabbits (12-week-old, 2–3 kg) by the Second Military Medical University Experimental Animal Center (China) were used for in vivo studies for MRI test. They were divided randomly and equally into ATF group and control group. Briefly, under general anesthesia with pentobarbital sodium, a 1.5-cm incision was made in the skin, then the L6 spinous process, adjacent interspinous and supraspinous ligament was removed by rongeur (Fig. 1b). In ATF group, the ATF linear material was used as substitute material to fix between spinous process of L5 and L7 with a continuous suture (4-0, Mersilk, Ethicon, Johnson & Johnson) (Fig. 1c). Then, the bilateral paraspinal muscle was sutured on the ATF linear material (Fig. 1f). In control group, the bilateral deep fascia was sutured together with a continuous suture which was usually used for lumbar surgery (Fig. 1e).

#### Rat

Ten male SD rats (8-week-old, 200–220 g) by the Second Military Medical University Experimental Animal Center (China) were used for in vivo studies for ultrasound, electromyography (EMG) and histological test. The surgery process was similar to that in rabbit. The difference is the L5 spinous process, adjacent interspinous and supraspinous ligament was removed.

All animals were carefully observed complications were evaluated such as infection and bleeding. Animal husbandry, experimental procedures, and euthanization were performed according to animal ethics standards. Three months after the intervention, the morphology, volume and function of paraspinal muscles were tested via ultrasonic, MRI and Electromyogram test.

### Ultrasonic test

SD rats (*n* = 10) were sedated by intraperitoneal administration of 10% chloral hydrate solution (3 ml/kg), and the both hind limbs extend back naturally in prone position. Bilateral paraspinal muscles were tested through ultrasound (L12-5/THYFC, PHILIPS, Netherlands) with high frequency probe (5–12 MHz). The longest diameter lines of the paraspinal muscle were measured on the cross-section area in ventral-dorsal and cephalococcal plane. The normal animal without any intervention was used as reference group.

### MRI scan

New Zealand rabbits (*n* = 10) were sedated by intraperitoneal administration of 10% chloral hydrate solution (3 ml/kg), then underwent full body MRI scan (ACHIEVA 1.5T, PHILIPS, Netherlands) set with TR2500, TE105, slice thickness 4 mm, NEX 2–4, array 256 × 256. Morphology of paraspinal muscles and signal changes between muscle group and lamina were observed. The normal animal without any intervention was used as reference group.

### EMG test

SD rats (*n* = 10) were used for the EMG test, of which the process was divided into two phases: rest state and waking state. In the resting state, SD rats were sedated by intraperitoneal administration of 10% chloral hydrate solution (3 ml/kg), and the fixed on the operating table. The low back area was disinfected with povidone iodine. The needle was pierced into the paraspinal muscles on both sides of the surgical site and the amplitude, time phase and frequency were recorded by electromyograph (Alpine bioMed Aps, Denmark). Similarly, in the awake state, the head was fixed and the same area was tested. The normal animal without any intervention was used as reference group.

### Histological test

**Fig. 2:**
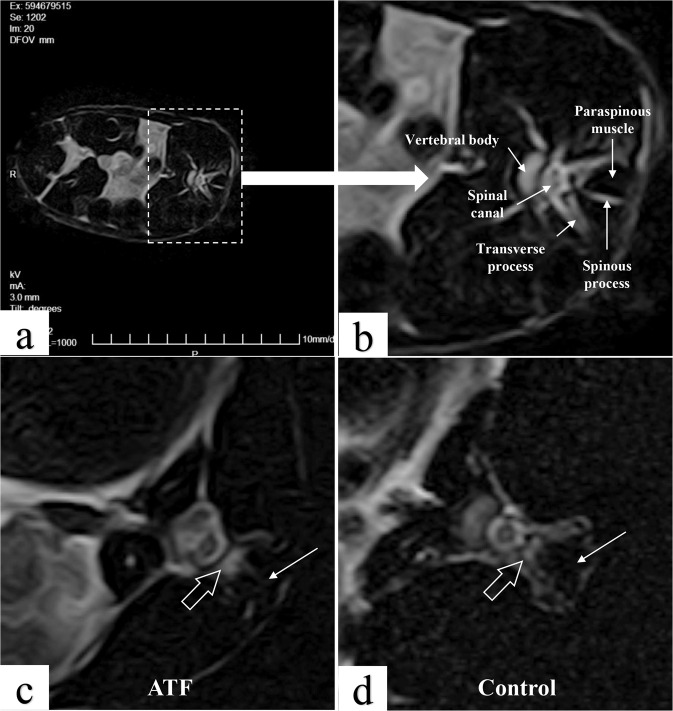
MRI scan results of paraspinal muscle for volume evaluation in rabbits. **a** Magnified figure of MRI on lumbosacral spine of normal New Zealand white rabbit; **b** MRI structure of normal L5; **c** MRI results showed regular shape and basically uniform signal of paraspinous muscle in ATF group; **d** MRI results showed the irregular shape and uneven signal of the boundary of paraspinous muscle in control group. The white arrow indicates the paraspinous muscle, and the white transparent arrow indicates the space between the paraspinous muscle and the lamina

The SD rats (*n* = 10) were euthanized 3 months after the surgery, and the specimens (muscle-ATF-muscle complex in ATF group and muscle-muscle complex in control group) were harvested and fixed with 4% paraformaldehyde solution for 24 h, then decalcified in 5% formic acid for 24 h and dehydrated in graded ethanol solutions (Fig. 2). Then, the specimens were embedded in paraffin and sectioned with microtome (RM2235, Leica, Germany). The sections were polished stained with hematoxylineosin (HE) and Masson trichrome to observe the structure of paraspinal muscles, the healing of muscle and ATF material. Immunohistochemical test, including Collagen I antibody and immune inflammation factor such as CD11B, CD11C, CD3 and CD68, was used to observe the changes of collagen composition and immune rejection reaction around the ATF material.

### Statistical analysis

All data were analyzed by using SPSS software (SPSS Statistics 16, Chicago, USA). Quantitative data were expressed as mean ± standard deviation. All statistical significance was determined by one-way analysis of variance (ANOVA) or independent Student’s t test Statistical significance was considered when *P* < 0.05.

## Results

### Ultrasonic test

The average weight of rats between two groups was not significantly different (267.8 ± 7.5 g vs 268.4 ± 8.5 g). The sum of the longest diameter lines of the paraspinal muscle on the cross-section area in ventral-dorsal and cephalococcal plane was set as the indicator of the muscle mass change. The measurement data of the two groups were shown in Table 1. In control group, the mean sum of the cross-sectional area of the bilateral paraspinal muscles is 2.05 ± 0.16 cm^2^, while in ATF group, the mean value was 2.37 ± 0.24 cm^2^, and the difference between the two groups was statistically significant. After reconstruction with the ATF linear material, the cross-sectional area of the paraspinal muscle group was larger than that of control group (Table 1, *P* = 0.037 < 0.05).

**Table 1:** Measurement data of the two groups of the paraspinal muscle on the cross-section area

Group	1	2	3	4	5	Mean	Standard deviation	*P* value
Control (cm^2^)	1.99	1.83	2.05	2.16	2.23	2.05	0.16	0.037
ATF (cm^2^)	2.19	2.23	2.27	2.39	2.78	2.37	0.24	

### MRI scan

Compared with normal New Zealand rabbit paraspinal muscle, MRI scan results showed the irregular shape and uneven signal of the boundary of paraspinous muscle, and high signal changes between the paraspinous muscle and the lamina in control group. In the ATF group, MRI showed regular shape and basically uniform signal of paraspinous muscle, and there are a few areas of high signal between the paraspinous muscle and the lamina (Fig. 2).

### EMG test

**Fig. 3:**
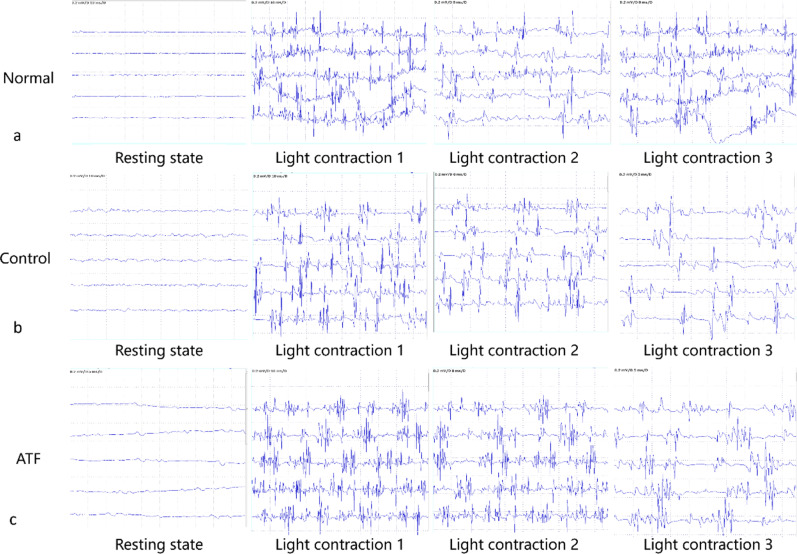
EMG examination results of SD rats for paraspinal muscle function test. **a** EMG results of normal SD rats in resting state and awake light contraction state; **b** Control group EMG results showed more interference waves on the baseline without pathological wave and no low amplitude or shortened phase, but frequency of evoked potential in control group was lower than ATF group; **c** ATF group EMG examination showed straight baseline without pathological wave manifestations

In the resting state, EMG examination showed straight baseline without pathological wave manifestations in ATF group, which was similar with the normal one. While in control group, EMG showed more interference waves on the baseline without pathological wave. In the awake state, compared with the normal rats, there was no low amplitude or shortened phase in both groups, but frequency of evoked potential in control group was lower than ATF group and normal rats (Fig. 3).

### Histological test

**Fig. 4:**
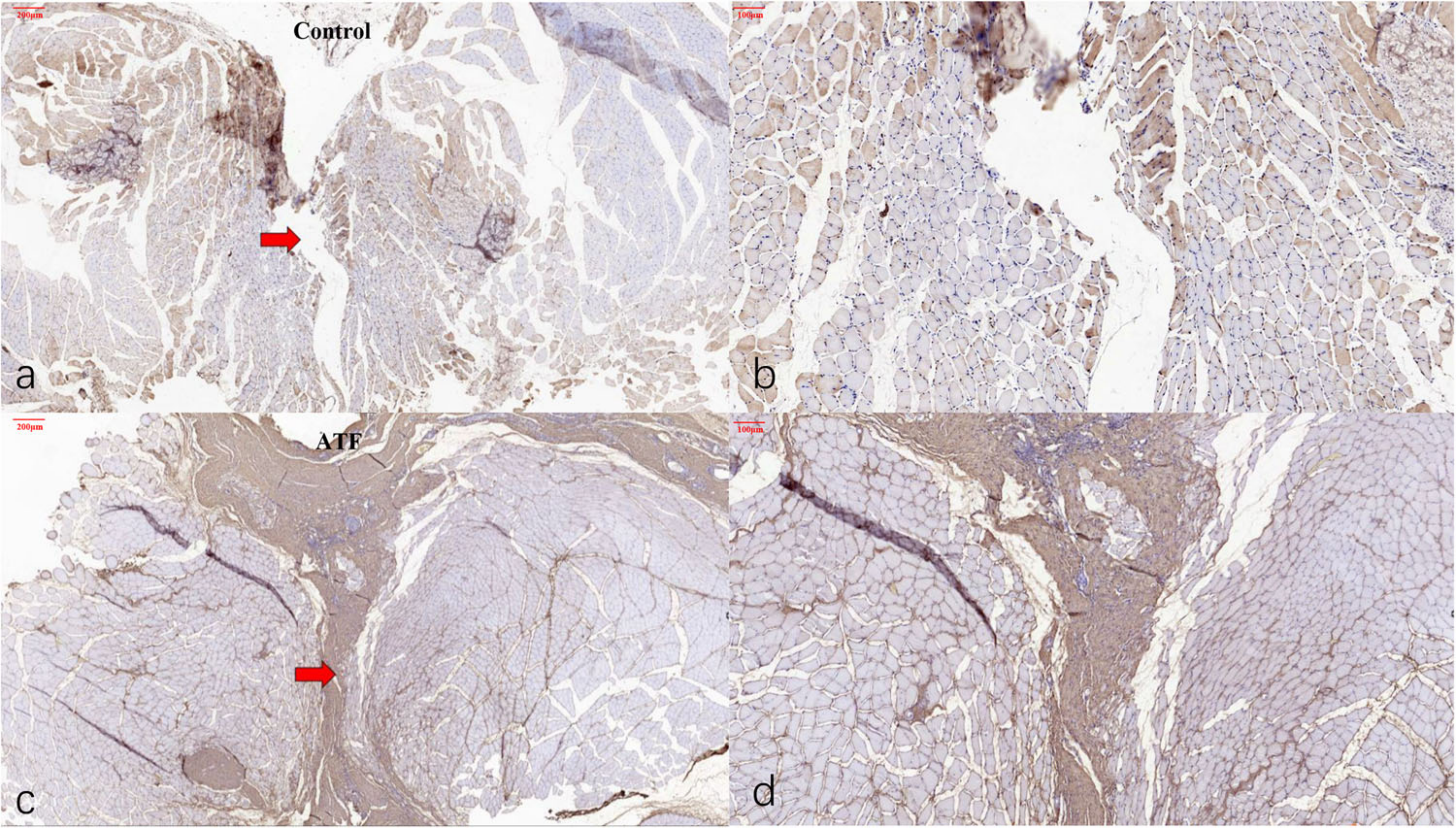
Immunohistochemical staining results of SD rats for Collagen I antibody of specimens in ATF and control group. **a**, **b** Histological test showed obvious gaps between the bilateral paraspinal muscles in control group. Red arrow indicated the tissue gap (×62 and ×124); **c**, **d** The bilateral paraspinal muscle and the collagen fibers between them were well healed, and the tissue structure of the bilateral muscle was intact in ATF group. Red arrow indicated the ATF linear material (×74 and ×148)

Three months after the operation, the specimens in control group showed obvious gaps between the bilateral paraspinal muscles. In ATF group, histological test showed that the bilateral paraspinal muscle and the collagen fibers between them were well healed, and the tissue structure of the bilateral muscle was intact (Fig. 4). The type I collagen immunohistochemical staining of the ATF linear material showed positive manifestation, with more fibroblast-like host cells migrating inside.

**Fig. 5:**
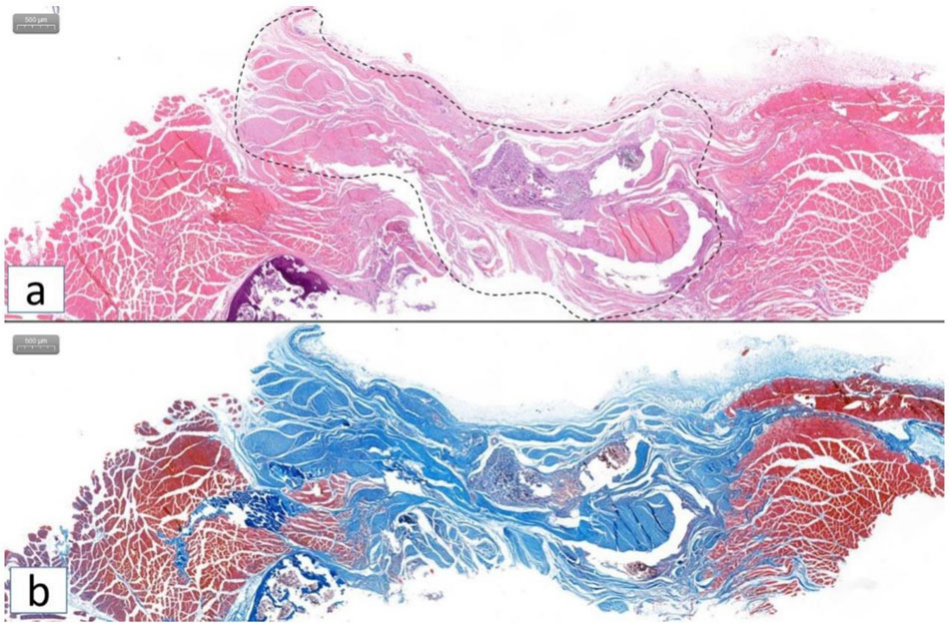
HE and Masson staining of specimens in ATF group in SD rats. **a** HE staining and **b** Masson stating showed that the collagen fiber structure and the muscle tissue structure were intertwined, and there was no obvious tissue gap, suggesting good tissue healing. Dashed line area indicates the ATF linear material

HE and Masson staining showed that the collagen fiber structure and the muscle tissue structure were intertwined, and there was no obvious tissue gap, suggesting good tissue healing (Fig. 5).

**Fig. 6:**
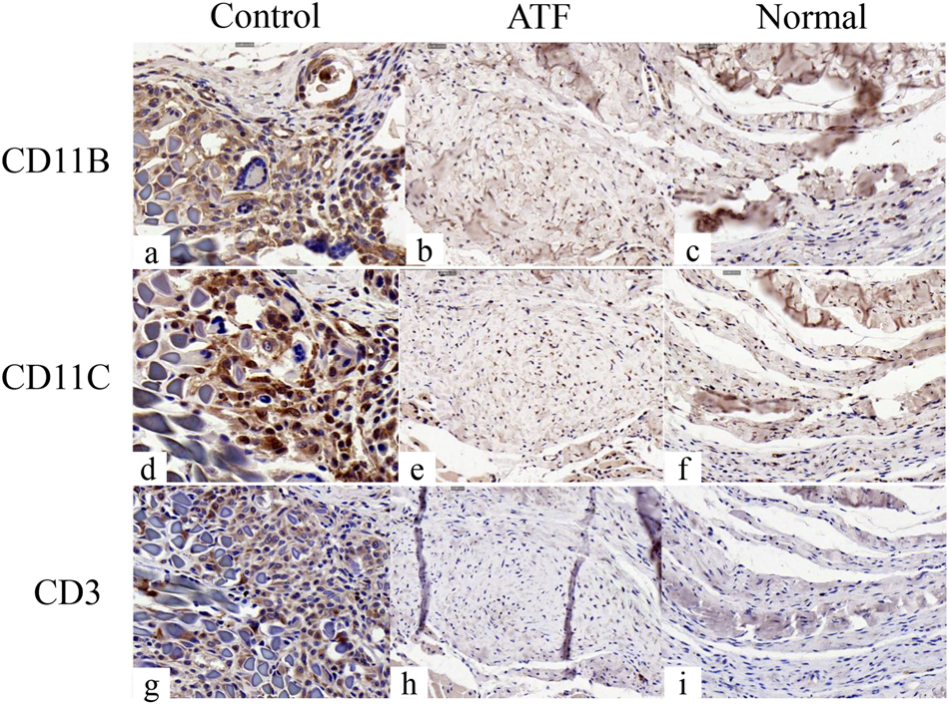
Immunohistochemical staining results for immune inflammation factor of specimens in ATF and control group in SD rats. **a**–**f** Immunohistochemical test of neutrophils, monocytes, macrophages, NK cell markers (CD11B and CD11C). Suture used in the surgery showed strongly positive (**a**, d × 700), ATF material showed positive (**b**, e × 500), and normal low back fascia collagen fibers showed positive (**c**, f × 500); **g**–**i** Immunohistochemical test of T cell markers (CD3). Suture used in the surgery showed strongly positive (g × 700), ATF material showed negative (h × 500), and normal low back fascia collagen fibers showed negative (i × 500)

Compared with SD rats’ own normal low back fascia collagen fibers, neutrophils, monocytes, macrophages, NK cell markers (CD11B and CD11C) were all positive, and T cell marker (CD3) was negative in ATF group. But the suture used in the surgery was positive for all four markers. These results suggest that at 3 months after surgery, the immune rejection of ATF linear material is significantly lower than that of control group (Fig. 6).

## Discussion

The lumbar paraspinous muscle is of particular importance for the maintenance of stability and normal movement of spine [5, 14]. Long incisions, extensive detachment of paraspinal muscles from the spinal processes, screws implant through multifidus muscle, and subsequently prolonged wide retraction can cause varying degrees of damage to paraspinous muscle, resulting in ischemic necrosis and denervation of the musculature in posterior lumbar surgery [7, 12, 15]. Indeed, minimally invasive approaches could reduce the injury of lumbar paraspinous muscle [8], but it is not always possible in the surgical treatment of spinal pathology [16–18]. In order to obtain sufficient decompression for nerves in the spinal canal, it is inevitable to detach the paraspinous muscles and remove the attachment structures of the paraspinous muscles such as spinous processes, interspinous ligaments, and lamina. Excessive retention of the attachment structure of the paraspinous muscles will hinder the decompression operation. Preservation of the attachment structure is important in preventing the paraspinous muscles from degeneration and denervation. Kota et al. performed a new procedure, the lumbar spinous process–splitting laminectomy, involving exposure of the lamina by longitudinally splitting the spinous process into two halves, leaving its muscular and ligamentous attachments undisturbed. After decompression, each half of the split spinous process is reapproximated using a strong suture to preserve the supra- and interspinous ligaments [19]. Neil et al. proposed the surgical technique of reattachment and repair of lumbar multifidus to restore paraspinous muscle integrity to minimize postoperative low back pain [6]. Kenichi developed a novel surgical approach, hemilateral split-off of the spinous process, to the lumbar spine in which the attachment of the paravertebral muscles to the spinous process was preserved [20]. If the attachment structure of the paraspinous muscles removed for decompression can be reconstructed, the paraspinous muscles could be re-fixed to the reconstructed attachment structure, which may prevent the continuous contracture and help the function recovery of the incision muscle, thereby reducing the incidence of low back pain after lumbar posterior surgery. In this study, attachment defect model of paraspinous muscles in rabbits and rats was successfully established, and the ATF linear material was used in the reconstruction of muscle attachment structure.

Muscle degeneration is characterized by a decrease in the size of the muscle and/or an increase in the amount of fat deposits. MRI is useful for measuring the muscle mass and degeneration degree of paraspinous muscles, which has been reported in patients with chronic back pain [21–24], so does ultrasound [25–27]. In this study, ultrasound showed the cross-sectional area of the paraspinal muscle group was larger than that of control group after reconstruction with the ATF linear material, which suggested that reconstruction with the ATF linear material might prevent paraspinal muscle atrophy after posterior lumbar surgery. In MRI results, paraspinous muscle in ATF group possessed regular shape and uniform signal, and abnormal signal between the paraspinous muscle and the lamina was shown in control group. This was suggested that the ATF linear material could maintain the shape of the paraspinous muscle, and there was no obvious inflammatory reaction after the reconstruction. EMG also showed similar results. Under the same stimulus intensity, the frequency of the EMG wave decreased significantly in control group, and the weakened response may be related to the atrophy of the paraspinal muscles without rebuilding the attachment structure. After the attachment structure of the paraspinal muscles is reconstructed, the muscle volume of the bilateral paraspinal muscles is greater than that of the non-reconstructed group. The phenomenon suggested the paraspinous muscle of rats in control group was hypoergic and this presumably might be related to the paraspinal muscle atrophy.

Low immunogenicity plays an important role in the tissue transplantation. In our previous study [13], the ATF linear material obtained by the proposed mechanical method is 50–130 μm in diameter and 20–80 mm in length, which showed good cytocompatibility and histocompatibility. ATF were derived from the extracellular matrix of bovine tendons, where the primary component is type I collagen [28]. During the decellularization processes, collagen fibers are fully exposed to decellularization reagents. The proposed decellularization process removed cells and retained the structure of native spiral-shaped fibrils, which is one reason why ATF exhibits acceptable cytocompatibility. The antigenicity of allografts and xenografts is primarily due to cells and proteins in transplanted tissue [29–32]. Collagen I is principally the main reason for the antigenicity of ATF. The basic collagen structure is conserved among species; therefore, it possesses minimal inherent immunogenic potential [33–36]. In addition, deep freezing [37, 38] and lyophilization [39] are the main ways to reduce the immunogenicity of the allograft. In our study, immunological rejection related cells such as neutrophils, monocytes, macrophages and NK cell, the markers (CD11B and CD11C) were all positive, and T cell marker (CD3) was negative in ATF group, but for suture, all the four markers were strongly positive. The results demonstrated that the ATF linear material had good heterogeneous histocompatibility and small immune rejection.

## Summary

In conclusion, defect of spinous process, interspinous and supraspinous ligament was established on lumbar spine in rabbit or rat and ATF linear material was implanted successfully in this study. Reconstruction of the attachment structure of paraspinous muscles with ATF linear material could maintain the morphology, volume and function of paraspinal muscle, which may reduce the postoperative low back pain. In addition, this work suggested ATF material has the potential to be used to manufacture personalized ligaments and other tissue engineering scaffolds.

## Supplementary information

Supplementary Information

## Data Availability

The datasets used during the current study available from the corresponding author on reasonable request.
