# Supplementary material for: Lyophilized bovine acellular tendon linear fiber material for the reconstruction of attachment structure of paraspinous muscles: an animal in vivo study
Source: J Mater Sci Mater Med. 2022 Dec 3;33(12):79. doi: 10.1007/s10856-022-06701-3 (PMC9719447; doi:10.1007/s10856-022-06701-3)
Supplement: Supplementary file 1 — Supplementary Information [file 10856_2022_6701_MOESM1_ESM.docx]

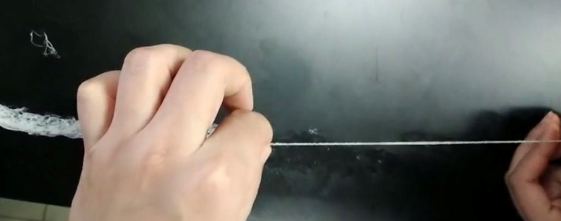


Supporting Information 1. Unidirectional twisting into linear decellularized tendon fiber material after wetting.


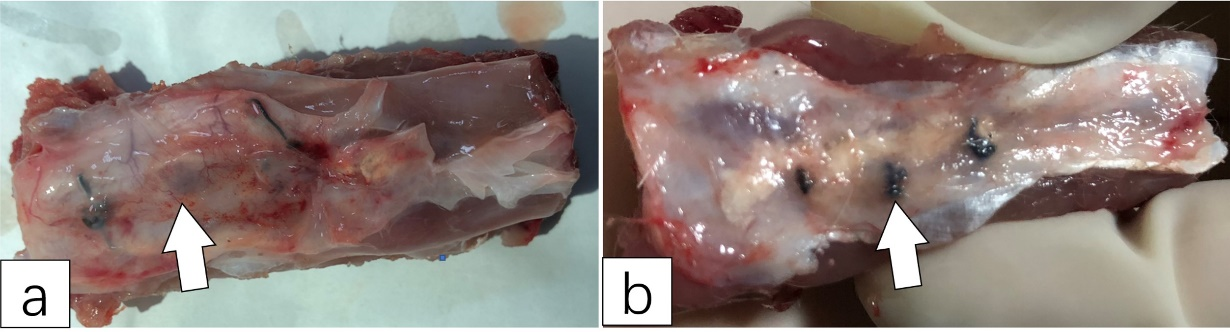


Supporting Information 2. Specimens harvested from rat. (a) specimen of the attachment structure of the paraspinal muscle group was reconstructed; (b) the specimen of the attachment structure of the paraspinal muscle group was reconstructed by AFT linear material. The white arrow points to the detection area of histopathological section.
